# Study on the Refusal of Vaccination against COVID-19 in Romania

**DOI:** 10.3390/vaccines10020261

**Published:** 2022-02-09

**Authors:** Flavius-Cristian Mărcău, Sorin Purec, George Niculescu

**Affiliations:** Faculty of Educational Sciences, Law and Public Administration, Constantin Brâncuși University of Târgu Jiu, 210185 Târgu Jiu, Romania; sorin.purec@utgjiu.ro (S.P.); george.niculescu@utgjiu.ro (G.N.)

**Keywords:** vaccination, COVID-19, pandemic, medical education, vaccination refusal

## Abstract

The refusal to be inoculated with the anti-COVID-19 vaccine by a part of the Romanian population becomes a barrier against controlling and stopping this particularly infectious virus. The rapid evolution of COVID-19 vaccines has created confusion regarding health and safety. Many Romanian citizens refuse vaccination because of fears generated by uncertainties based on information obtained from fake news. At the present moment, January 2022, Romania has one of the lowest vaccination rates in the European Union, below 45% of the total population. In our study, we want to identify the determining factors behind the refusal of vaccination, offering a sociological analysis that, we hope, will help to understand this phenomenon. The analysis revealed that 81% of the respondents trust the mandatory vaccines under the national scheme and 57.3% trust the optional ones other than the anti-COVID-19 vaccines (like Rotavirus, Hepatitis A and B, Influenza, Meningococcal, Pneumococcal, etc.) and have less confidence in the anti-COVID-19 vaccines. The study also reveals a very high percentage of respondents who trust fake news claims.

## 1. Introduction

The COVID-19 pandemic is an ongoing dynamic due to the mutations that SARS-CoV-2 virus is undergoing. The world is currently experiencing the fifth wave [1,2,3] due to the Omicron variant, and the daily reported number of infected people is reaching alarming levels.

The COVID-19 vaccines are currently the main viable weapon [4] in the fight against the pandemic, but the low vaccination rate in some countries [5] such as Romania and Bulgaria, compared to the rest of the European Union, puts extreme pressure on the medical system in such countries. The Romania’s vaccination policy has not succeeded in persuading people to get vaccinated against COVID-19 in large numbers. Thus, a low vaccination rate among the population leads to a high number of infected people, people admitted to intensive care units (ICU) and dead people reported daily by the State.

From the moment of the approval and availability of COVID-19 vaccines [6], Romania has had an exponential increase in cases and, at this moment, the fifth wave has increased the number of infected people [7]. However, the Romanian population is still reluctant to vaccinate against the disease.

The measures applied to stop the spread of the virus such as physical distancing, body temperature measurement, quarantine of infected people and their contacts, as well as limitation of travelling within a certain time frame, have protected, a little, rightly so, the community and decreased the pressure on the medical system suffocated by the large number of cases [8].

**Figure 1:**
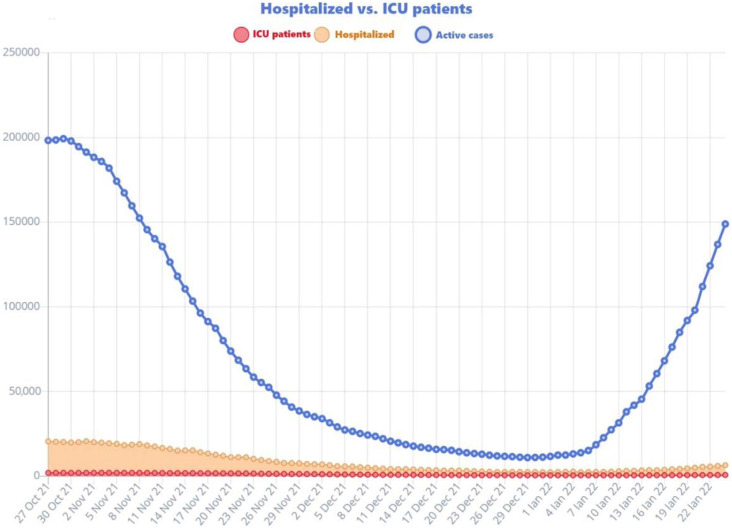
Representation of hospital admissions, admissions to intensive care wards and deceased during this period [10].

At the time of the fifth wave of the COVID-19 pandemic, Romania went through an unprecedented medical crisis. The medical system was approaching collapse. The healthcare system came close to collapse during the fourth wave [9], the number of intensive care beds dedicated to COVID-19 patients were almost entirely occupied and the increasing number of deaths reported daily reached alarming levels, as can be seen from the following chart (Figure 1).

The number of the people who have died relative to the number of infected raises a big question mark if we consider a comparison between Romania and the rest of the European Union.

The insufficient vaccination in Romania does nothing but prolong the COVID-19 pandemic and cause a lot of deaths, as happened in the fourth wave, compared to states that have a high vaccination rate. A part of the population shows unjustified resistance when the discussion about vaccination arises, regardless of the type of vaccine. The lack of a minimum medical culture, disinterest in science and the rejection of scientific arguments turns the country into a potential laboratory of mutations due to the huge number of cases.

In this study, we aim to highlight the factors underlying hesitation or refusal of vaccination. We want to find a common denominator for this “fear of the vaccine”, determined by conspiracy theories, lack of medical education or fear of novelty. In addition,, COVID-19 outbreaks can be controlled or stopped by vaccinating residents, but the rejection of the vaccine raises serious problems and moves the pressure onto the medical system in Romania, which will inevitably collapse at some point. Therefore, considering that Romania has a very low vaccination rate, it is important to show why people refuse vaccination against COVID-19. The reasons for the rejection of COVID-19 vaccines by a part of the population can shape a serious vaccination policy that can lead to a significant increase in the vaccination rate. Thus, the problems of hesitation and rejection of vaccines can be overcome and the pandemic can end. The acceptance of vaccination against COVID-19 is a very important step towards the control of the outbreaks of COVID-19 in the community, the decrease of hospital admissions and deaths.

This study aims to provide scientific research on the reasons for rejecting vaccination. The sufficient number of questionnaires completed allows us to draw such conclusions and, above all, to present the reasons that influence the acceptance of getting the vaccine.

## 2. Materials and Methods

### 2.1. Participants 

This study was conducted during the fourth wave of the COVID-19 pandemic, during the period when Romania was in second to last place in the ranking of EU states in terms of vaccination rate, November 2021.

We analyzed a number of 650 questionnaires, completed by Romanian citizens with permanent residence in Romania who have not been vaccinated so far. The respondents in the target group are people who have been included in the following categories:

▪ age from 16 years to 65+;
▪ permanent residence in rural or urban areas;
▪ different levels of education;
▪ have not been vaccinated against COVID-19;
▪ have had, or have not had the COVID-19 disease;
▪ permanent residence in Romania.

All participants were informed about this study and their participation was voluntary, with no elements of coercion.

### 2.2. Procedure

Participants were given a questionnaire created on the Google Forms platform, allowing them to distribute and complete it online through the web browser.

The questionnaire was anonymous and created without containing elements to identify the respondents.

The questionnaire was posted on various social media platforms and blogs with a large number of visitors per day.

### 2.3. Measurements

The questionnaire was designed in two sections, as follows:

1. The first section contains socio-demographic elements and questions on the acceptance/non-acceptance of vaccines included in the national mandatory vaccination scheme; acceptance of optional vaccines (Rotavirus, Hepatitis A and B, Influenza, Meningococcal, Pneumococcal, etc.);

2. The second section, except for a few sentences, contains statements from the area of fake news. We have chosen to note whether the combination between the refusal of vaccination and information from sources classified in the category of fake news can be valid.

### 2.4. Statistical Analysis of Data

The analysis and processing of the data has been carried out with the help of Excel, part of the Microsoft Office Professional Plus 2019 package, installed on a computer with a Windows 11 Professional operating system.

The data have been processed and interpreted from a synthetic and analytical point of view. The weightings were made according to age, level of education and residence, comparing the results in order to understand the differences in vision. In addition, the indicators aimed at rejecting/accepting the mandatory and optional vaccines (other than anti-COVID-19) were taken into account to see if there is reluctance only about the anti-COVID-19 vaccines or a vaccination opposition. The answers provided in the last part of the questionnaire had a very important role in determining the level of trust given to the information classified in the sphere of fake news.

As for the analysis of data extracted from the open question “What made you decide not to get vaccinated?”, the answers were gathered in their entirety and the data processing was executed manually, as AI-based software programs proved a rudimentary and inconclusive processing in some cases.

## 3. Results

This study includes the analysis of 650 valid answers, provided through the applied questionnaire. The socio-demographic data of the participants are shown in Table 1.

### 3.1. Trust in Doctors and the Medical System in Romania

**Figure 2:**
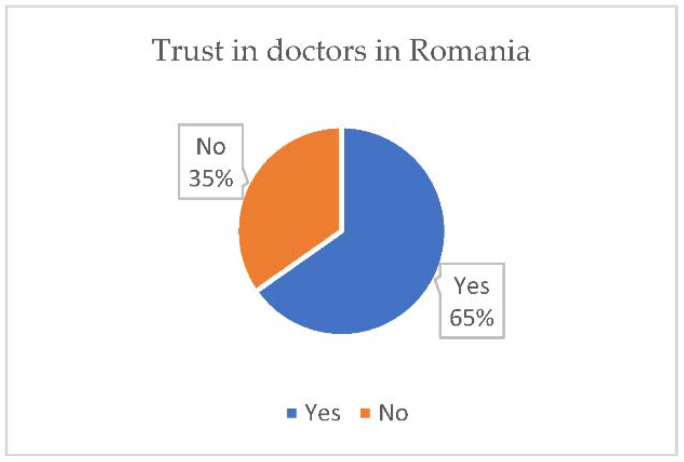
Trust in doctors in România.

**Figure 3:**
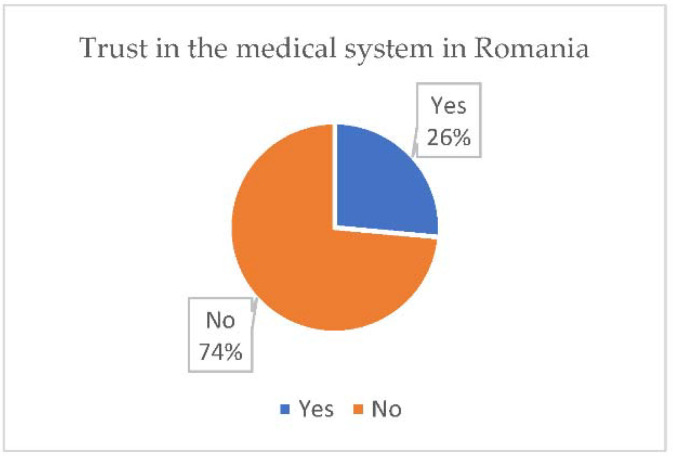
Trust in the medical system in România.

From the extracted data, there is an increased trust of the respondents regarding the doctors in Romania (Figure 2), but, as far as the medical system is concerned, the trust is significantly lower, indicating a rather low trust in the state institutions (Figure 3).

### 3.2. Trust in Vaccines Other Than Anti-COVID-19 Vaccines

Regarding the degree of trust given to the mandatory vaccines included in the national vaccination scheme, 81% of the respondents voted in favor of them and 57% in favor of optional vaccines other than those against COVID-19 (like Rotavirus, Hepatitis A and B, Influenza, Meningococcal, Pneumococcal, etc.).

The respondents did not agree to inoculate with one of the anti-COVID-19 vaccines approved in Romania even if 94.9% of them are vaccinated with the vaccines included in the national vaccination scheme of the population.

To the open question “What is the reason why you did not get vaccinated?”, from the multitude of arguments and personal formulations, we extracted the most common ones and grouped them into 12 categories, leaving a significant percentage of unclassifiable answers, according to Table 2.

### 3.3. Trust in Fake News

The second part of the questionnaire contained a set of statements, most of which were classified as “fake news”. The resulting data are presented in Table 3.

## 4. Discussions

In this study, conducted during the fourth wave of the pandemic, we set out to observe the reasons behind the refusal of vaccination against COVID-19. Although Romania is going through a very difficult period in terms of the number of infected and dead reported daily, a part of the population categorically refuses vaccination. The sample comprises 650 participants divided into 411 women and 239 men, and we consider that gender differences do not impact the analysis and conclusions of our study. Both genders gave roughly similar reasons for non-vaccination decisions and provided similar or close scores for “fake news” allegations.

We found that, out of the total number of participants, 33% have officially had the disease caused by the novel coronavirus or had at least one positive test, which excludes, on their part, the contestation of the existence of the disease. Therefore, the analysis was carried out starting from the idea of trust in the vaccine.

In order to draw some viable conclusions, we set out to follow the degree of trust that the participants give to the medical system and doctors in Romania. In addition, 74% of the respondents do not trust the medical system and 34.7% do not trust in doctors. We note that the distrust in the medical system is significantly higher compared to that regarding doctors, this being possible as a result of the problems that the system has encountered in recent years such as lack of medicines, lack of equipment, lack of modern constructions, poor infrastructure, hospital fires, etc. [11,12,13,14].

The trust that the participants have in the national vaccination scheme and optional vaccines [15] other than those against COVID-19 (like Rotavirus, Hepatitis A and B, Influenza, Meningococcal, Pneumococcal etc.) has an increased rate [6] compared to Romania’s vaccination rate against COVID-19 (below 38%). Thus, 81% of respondents trust the vaccines included in the national vaccination scheme, while optional vaccines enjoy the trust of 57.3%. However, 94.9% of the study participants said they had been vaccinated with the vaccines included in the national vaccination scheme.

Regarding the open question “What is the reason why you did not get vaccinated?”, the participants argue their decisions based on the lack of trust in the anti-COVID-19 vaccines (16.7%), considering that these are true experiments that are tested on the world population (12.6%), despite the research that demonstrates the effectiveness of vaccines against the SARS-CoV-2 virus [16,17,18,19].

However, a large proportion of respondents refuse vaccination because of immediate side effects (9.2%), medium and long-term side effects (7%) and the lack of sufficient evidence of available vaccines (6%).

Some of the respondents do not consider the anti-COVID-19 vaccination to be beneficial since they have had the disease and developed antibodies (4.1%). The disease can be contracted by vaccinated people (5.6%), but the vaccines together with the antibodies formed from the disease lead to a higher titer of protective antibodies [20] and reduce the risk in case of a new infection [21].

Some participants (5.3%) question the responsibility for any side effects that may occur following the inoculation of the vaccine, regardless of its type, especially since neither the pharmaceutical companies nor doctors or government officials assume any responsibility. The patient is obliged to sign a declaration assuming any adverse effects of the vaccine.

In addition to those mentioned above, there are participants who consider that vaccination is not necessary since they are healthy and have no comorbidities (4.9%) or participants (4%) who, according to the extracted data, have a medical recommendation not to get vaccinated against COVID-19. Regarding healthy people, there are studies that reveal that the new coronavirus is a life-threatening disease in itself that can also kill people of any age without comorbidities [22,23].

We presented the arguments of unvaccinated people from rural areas and those from urban areas in Table 4.

Regarding the trust that the participants have in the statements classified in the category of “fake news” (Table 3), the results are worrying given that there are no scientific arguments which can be supported. Thus, 47% of the participants believe there is a world occult organization that controls the world and wants to reduce the population of the Earth (44.1%). The interpretation of these percentages leads us to a conspiracy scenario that almost 30% of Romanians believe (we recall that, in Romania, 62% of the population is unvaccinated, and 47% of them, according to our research, believe that there is a secret world organization that manipulates us, which brings the percentage of these followers of the conspiracy theory to 29.1% per country).

In addition, 33.2% believe that doctors are paid to inoculate a vaccine that would help reduce the Earth’s population, and 24.9% are sure that people who have chosen to get the COVID-19 vaccine will die in the coming years from the inoculated substances. Worryingly, 40.9% of people believe that COVID-19 vaccines lead to infertility or death. In addition, 38.1% say the vaccines were created to reduce the aging population, while 13% believe in the conspiracy theory that it is intended to implant a microchip through the vaccine.

The participants’ confidence in the existence of the COVID-19 pandemic is questioned by 34.4%, and 49.8% consider that vaccination does not want to eradicate COVID-19.

This scenario is worrying because the percentage of unvaccinated people in Romania amounts to 59%, out of which few people are unvaccinated for relatively objective reasons. The anti-vaccine movement is closer to the Orthodox Church, which has not shown conclusive signs that it urges vaccination, linking the people who manifest against vaccines to divine inspiration and grace. Images of crowds of thousands of believers protected by God alone, receiving communion with the same teaspoon from a bishop in the midst of a restricted pandemic and helplessly witnessed by law enforcement, can hardly be forgotten [24,25,26]. Equally difficult to be forgotten is the priest, who, in the church during the religious service, recommended that the parishioners not be vaccinated because their tails or scales will grow [27], referring to the genetic changes produced by the vaccine. To resume, almost half of those who refuse to be vaccinated consider themselves a kind of heroic apostle of the human race fighting for its purity with a powerful, unnamed manipulative occult organization, enriched by the sale of vaccines that will lead to genetic modification of the species.

Almost the entire Romanian population was vaccinated with the so-called “mandatory” sera, inoculated at young ages, with rare expressed oppositions from parents, who perceived them as a regular treatment. COVID-19 vaccines come with a novelty: optionality. There are several options and people can choose one of them. There are even two types, the classic non-replicative viral vector-based vaccine, and the messenger RNA-based vaccine. People usually do not have a clear understanding of how each of these two types works, but different research studies have revealed views on each. Our study indicates that mistrust is more likely to be directed towards the new type of vaccine, the messenger RNA-based type, which was also the first type of vaccine approved in Romania, showing that the reluctant people who finally got vaccinated opted for one of the classic vaccines, i.e., Astra-Zeneca or Johnson & Johnson.

## 5. Research Limitations

Although our study has many strong points, being among the first studies of this type in Romania, there are some limitations. A first limitation is the lack of a question regarding people with comorbidities. Thus, we could have described much better why this category refuses vaccination. A second limitation is the percentage of people in rural areas that is lower than in urban areas. We believe that a higher number of people would have raised the confidence rates given to the “fake news”, as it is known that this class has a serious opposition when it comes to vaccination against COVID-19 [4,8,28,29]. 

## 6. Conclusions

The study highlights that the level of confidence in fake news is a matter of serious concern when it comes to vaccination against COVID-19.

Respondents showed a high degree of susceptibility to conspiracy theories, which led to their decision not to accept available COVID-19 vaccines. Although their concern about the new generation of messenger RNA-based vaccines is higher, it turns out that the rest of the COVID-19 vaccines cause just as much concern among participants.

The general vision of this large minority of the unvaccinated people is that there is a secret occult world organization that manipulates us with a non-existent disease because it wants to control the world and reduce the population through infertility or slow death, the favorite target being the elderly. The immorality of this global occult is high; it wants to enrich vaccine manufacturers not to eradicate the apparent disease. These vaccines will lead to genetic changes in humans in the future. Doctors who make the vaccines are part of this occult world; they are paid by this organization for their work. 

The lack of an elementary medical culture of the people and a lack of vaccination campaign of the state lead to such a scenario that creates a conspiratorial world to which more and more people adhere. We also believe that the spread of false information and conspiracy theories is not only mainly due to social media platforms and malicious websites [30,31,32,33,34], but also due to the traditional media that have induced panic, generally in less educated environments. In addition to the above, we agree that a pro-vaccination campaign, based on scientific information presented by specialists in the field of virology and infectious diseases, would lead to an increase in the acceptance of anti-COVID 19 vaccines among hesitant individuals.

## Figures and Tables

**Table 1:** Representation of the characteristics of the survey participants.

Features	Doctorate	Faculty	Masters	Secondary Education	High School Education	General Total
Feminine	10	214	90	9	88	411 (63.2%)
Rural	2	55	16	3	27	103 (15.8%)
Urban	8	159	74	6	61	308 (47.3%)
Masculine	9	112	60	8	50	239 (36.7%)
Rural	2	24	11	3	17	57 (8.7%)
Urban	7	88	49	5	33	182 (28%)
General total	2.9%	50.1%	23%	2.6%	21.2%	650 (100%)
Standard Error	0.002	0.043	0.019	0.001	0.016	*p* > 0.01
Standard Deviation	0.005	0.106	0.048	0.003	0.039	
Confidence Level (95%)	0.005	0.112	0.05	0.004	0.041	

**Table 2:** Data setting out the main reasons given by the persons when arguing for the non-vaccination decision.

Arguments	%
DISTRUST	
Distrust of COVID-19 vaccines	16.7
COVID-19 vaccines are an experiment	12.6
Vaccines have not been sufficiently tested	6
Too little information about COVID-19 vaccines	5.2
VACCINE SIDE EFFECTS AND REACTIONS	
Immediate side effects	9.2
Medium and long-term side effects	7
Government, doctors or vaccine manufacturers do not take responsibility for potential adverse effects of COVID-19 vaccines	5.3
OTHER REASONS	
Health problems that do not allow vaccination against COVID-19	4
Healthy people who believe that vaccination against COVID-19 is not necessary	4.9
People vaccinated against COVID-19 can contract the disease so the vaccine is useless	5.6
Those who have had COVID-19 think they do not need to get vaccinated because they are protected by antibodies	4.1
Restrictions imposed by the government on unvaccinated persons	5.8
Other reasons	13
Total	99.4 *

* the total percentage did not result in 100% due to the fact that the rounding was made to one decimal.

**Table 3:** The faction by the survey participants of the statements of the type “fake news”.

	Disagreement (1 and 2)		Uncertain (3)		Agreement (4 and 5)		*p*
Claims	N	%	N	%	N	%	
The COVID-19 pandemic is real	224	34.4	129	19.8	297	45.7	*p* < 0.05
There is a global secret society who wants to control the world	231	35.5	113	17.3	306	47	*p* = 0.01
COVID-19 vaccines are made to reduce the Earth’s population (infertility, death, etc.)	281	43.2	103	15.8	266	40.9	*p* = 0.01
Doctors are paid to inoculate a vaccine that would help reduce the Earth’s population	322	49.5	112	17.2	216	33.2	*p* > 0.01
People who have been vaccinated against COVID-19 will die in the coming years because of this	336	50.1	162	24.9	162	24.9	*p* = 0.03
Through the anti-COVID vaccine, it is desired to implant a CIP in the body	492	75.6	73	11.2	85	13	*p* < 0.05
Vaccination aims to kill the elderly	295	45.3	107	16.4	248	38.1	*p* > 0.01
Vaccination on a global scale aims to enrich vaccine manufacturers	198	30.4	96	14.7	356	54.7	*p* > 0.01
There is a world occult that wants to reduce the population of the Earth	263	40.4	100	15.3	287	44.1	*p* = 0.01
COVID-19 vaccination aims to stop the pandemic	324	49.8	154	23.6	172	26.4	*p* = 0.01
New messenger RNA-based vaccines produce dangerous genetic modifications	225	34.6	161	24.7	264	40.6	*p* < 0.05

**Table 4:** Data presenting the main reasons invoked by people from rural and urban areas when arguing the non-vaccination decision.

Arguments	Environment Urban	Environment Rural
	%	%
Distrust of COVID-19 vaccines	16.5	17.5
COVID-19 vaccines are an experiment	12.6	12.5
Immediate side effects	8.1	12.5
Medium and long-term side effects	6.7	8.1
Vaccines not sufficiently untested	6.1	5.6
Too little information on COVID-19 vaccines	4.2	8.1
People vaccinated against COVID-19 can contact the disease	5.7	5.6
The existence of antibodies due to the passage through the COVID-19 disease	4.4	3.1
Restrictions imposed by the government on unvaccinated persons	5.9	5.6
No one assumes the potential adverse effects of COVID-19 vaccines	5.3	5.6
Health problems that do not allow vaccination	4.2	3.1
I am a healthy person	5.3	3.7
Other reasons	14.4	8.7
	*p* > 0.01	
Total	99.1 *	99.7 *

* the total percentage did not result in 100% due to the fact that the rounding was made to one decimal.

## Data Availability

https://figshare.com/ndownloader/files/33988622 (accessed on 29 August 2021).
